# Prevalence of root caries among elders living in residential homes of Bengaluru city, India

**DOI:** 10.4317/jced.52682

**Published:** 2016-07-01

**Authors:** Balasubramanian Kumara-Raja, Gubbihal Radha

**Affiliations:** 1 BDS, Post Graduate Student. Department of Public Health Dentistry, V.S Dental College & Hospital, Bengaluru 560004; 2 MDS, Reader. Department of Public Health Dentistry, V.S Dental College & Hospital, Bengaluru 560004

## Abstract

**Background:**

Among the various oral ailments which have been observed in elderly, root caries is a significant one. Tooth loss is chief oral health-related negative variable to the quality of life in elderly and root caries is the major cause of tooth loss in them. It has been reported about a third of older population bears most of the root caries burden, so the present study aimed to assess the prevalence of root caries among older individuals residing in residential homes of Bengaluru city India.

**Material and Methods:**

Elderly individuals aged 60 and above, residing in residential homes of Bangalore city, were included in the study. The study participants filled a questionnaire regarding their demographic details and oral health habits. Root surface caries was recorded according to criteria described by Banting et al. and root caries was expressed in terms of the root caries index (RCI). The statistical analysis was performed using descriptive statistics and chi-square test. *P* < 0.05 was considered as statistically significant.

**Results:**

The prevalence of root caries was 46.4%. The root caries index was 15%. Statistically significant differences (*P* < 0.05) observed across gender, marital status, diet, socio-economic status, medication, method of cleaning and frequency of cleaning and were identified as significant predictors of root caries.

**Conclusions:**

The prevalence of root caries among institutionalized older people was high. Oral health policies and preventive measures are needed focusing on the special needs of this neglected and socioeconomically deprived population to improve their quality of life.

** Key words:**Elders, residential home, root caries.

## Introduction

Throughout the world, a demographic revolution is underway. The proportion of older people is growing faster than of any other age group. Approximately 600 million people are aged 60 years and over, and this number will double by 2025. By 2050, it will be 2 billion, 80% living in developing countries (1). In India the size of the elderly population has risen from 12.1 million in 1901 to approximately 77 million in 2001. According to official population projections, the number of elderly persons will rise to approximately 140 million by 2021. So this poses tremendous challenges to health and social policy planners, particularly because disease patterns will shift concurrently.

This increase in life expectancy with improvements in oral health status will ensure that many individuals will retain some or all their teeth into old age. But globally, poor oral health is evident in terms of high caries experience in both coronal and root surfaces and high prevalence rates of periodontal disease have been observed among older adults in recent times (1).

Among the oral ailments which are observed by dental practitioners in elderly, root caries is a significant one. Tooth loss is chief oral health-related negative variable to the quality of life in elderly and root caries is the major cause of tooth loss in them (2). It has been reported about a third of older population bears most of the root caries burden (3).

Thus root caries lesions was defined as soft, progressive, destructive lesions, either totally confined to the root surface or involving undermining of enamel at the cemento-enamel junction but clinically indicating the lesion initiated on the root surface (4).

Root surface caries is one of the significant oral health problems in the elderly, and incidence of root surface caries has been found to be one of the major risk factors for tooth loss in elderly.

Root caries is now been considered as a major public health problem for the elderly because there are three main interrelated arguments supporting this statement. Firstly, life expectancies at both birth and age 65 have been increasing markedly in industrialised societies. Secondly, there is ample evidence showing that periodontal disease increases with age due to its cumulative nature. Thus, most old adults may have some gingival recession and alveolar bone loss which shall predispose for root caries. Finally, improved oral health is causing the elderly to experience a higher retention of teeth, which implies an increased number of exposed root surface susceptible to caries (5).

Several epidemiological studies on root caries were conducted in the various parts of the country; however, only limited results from representative samples are available. Meanwhile, most oral health surveys conducted in India have been targeted at children and adolescents. Until now, only a few studies have reported on elderly population regarding their root caries experience. Therefore, data on root prevalence is needed for understanding and treating the problem. Moreover, India is experiencing significant changes in both the size and proportion of older adults, which is projected to continue, oral health research among elders becomes increasingly vital. Therefore, the present study aimed to assess the prevalence of root caries among older individuals residing in residential homes of Bengaluru city, India.

## Material and Methods

A descriptive cross – sectional study was done among 407 elder’s living at residential homes of Bengaluru city with an objective to assess the relationship between socio-demographic, oral hygiene habits and oral health behaviours with root caries.

All elders aged 60 years and above, who were willing to give the consent were included in the study. Elders who were edentulous, disoriented, bed ridden, uncooperative and not willing to fill the questionnaire were excluded from the study. The ethical approval was obtained before the commencement of study from the institution review board and the required official permission was taken from the chairman or trust member of each residential age homes. Written and verbal informed consent was obtained from the participants prior to them being interviewed and examined.

-Sampling method and sample size:

A total of 70 old age homes were registered under elder’s help line office of Bangalore city, among which 33 were run by private, 26 by trust, and 11 by society. A cluster sampling technique was employed in which old age was divided into three clusters and one cluster was taken using simple random sampling in which all the elder’s from private old age home were taken for the study.

Nicolau B *et al.* (6) in 2014 reported prevalence of root caries was to be 41.9% among individuals attending a rural health centre in Tumkur, South Karnataka, India. So for the present study prevalence of root caries was considered as 42% and population size of total elders residing in all old age home to be 1500. Finally, the minimum sample size required using this prevalence at 95% level of confidence and accepting a sampling error of 5% was calculated to be 306. So after meeting the inclusion and exclusion criteria a final sample of 312 elders were included for the study.

-Pilot study:

A pilot study was done among 50 elderly aged 60 years and above who visited the department of prosthodontics, V.S dental college and hospital for proper planning and execution of the main study. These subjects were not included in the main study. A face-to-face interview using a structured questionnaire was conducted to collect the following information: socio-demographic characteristics, use of medication, oral hygiene practices and oral health behaviour. A modified kuppuswamy scale, with readjustment of the per capita income to suit the present levels was used for classifying the individuals into one of the five socio-economic categories (7). The questionnaire was made in both English and Kannada language and the validity was checked by back translation method, involving blind re – translation into English. The validity of translation was verified by experts in both languages. The internal consistency reliability was checked using cronbach’s alpha co-efficient which was found to be 0.70.

-Study procedure:

The intraoral examination was carried out by calibrated examiner under natural light using plane mouth mirror, sickle - shaped explorers and periodontal probes. The teeth were neither cleaned nor dried before the assessment. However, food debris obscuring visual inspection was removed. No radiographs were taken. Root caries was recorded in a full mouth design, including the third molars. Root surface caries was identified using criteria described by Banting *et al.* (7). For identification of root caries, following criteria was for categorizing it into present or absent. 1) A discrete well-defined and discoloured soft area. 2) An explorer enters easily and displays some resistance. 3) The lesion is located either in the cement-enamel junction or wholly on the root surface. Root surface caries was recorded on exposed buccal/labial and palatal/lingual aspects of the roots of each tooth. Root caries was dichotomized into root caries present and root caries absent. Root caries was expressed in terms of the root caries index (RCI), which indicates the proportion of susceptible (exposed) root surfaces that are attacked by caries (filled and/or decayed root surfaces) [Katz, 1980].

**Figure 1:**
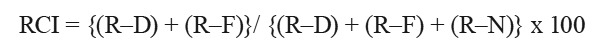
Root caries index (RCI).

Root caries index (RCI) was calculated for each subject according to Katz (4) as follows: (Fig. 1).

Where: R–D: Recession with decayed root surface R–F: Recession with filled root surface R–N: Recession with sound root surface. Intra-examiner variation in the clinical diagnosis of root surface caries was assessed by re-examining every 10th subject. The data was collected by a trained and calibrated examiner to ensure uniform interpretation and application of the criteria for the clinical assessment. In order to assess the consistency of the examiner, the intra – examiner reliability was done using kappa statistics which was found to be 0.82, reflecting a high degree of agreement in the observations.

-Statistical analyses

All the Statistical analysis was carried out using the statistical package for social sciences software, SPSS software version 19.0 (SPSS Inc., Chicago, IL, USA). *P* < 0.05 was considered as statistically significant. Chi-square tests were used for categorical variables.

## Results

A total of 312 elderly subjects with a mean age of 71.04 +/- 7.8 participated in the study. Among them 125(40.1%) were males and 187(59.9%) were females. In the study group 147(47.2%) were married and 165(52.8%) were unmarried, 67(21.4%) of them were illiterate, 163(52.2%) had a schooling experience, 82(26.4%) of them had attended college and none of the elder’s had a professional degree ( Table 1).

**Table 1:**
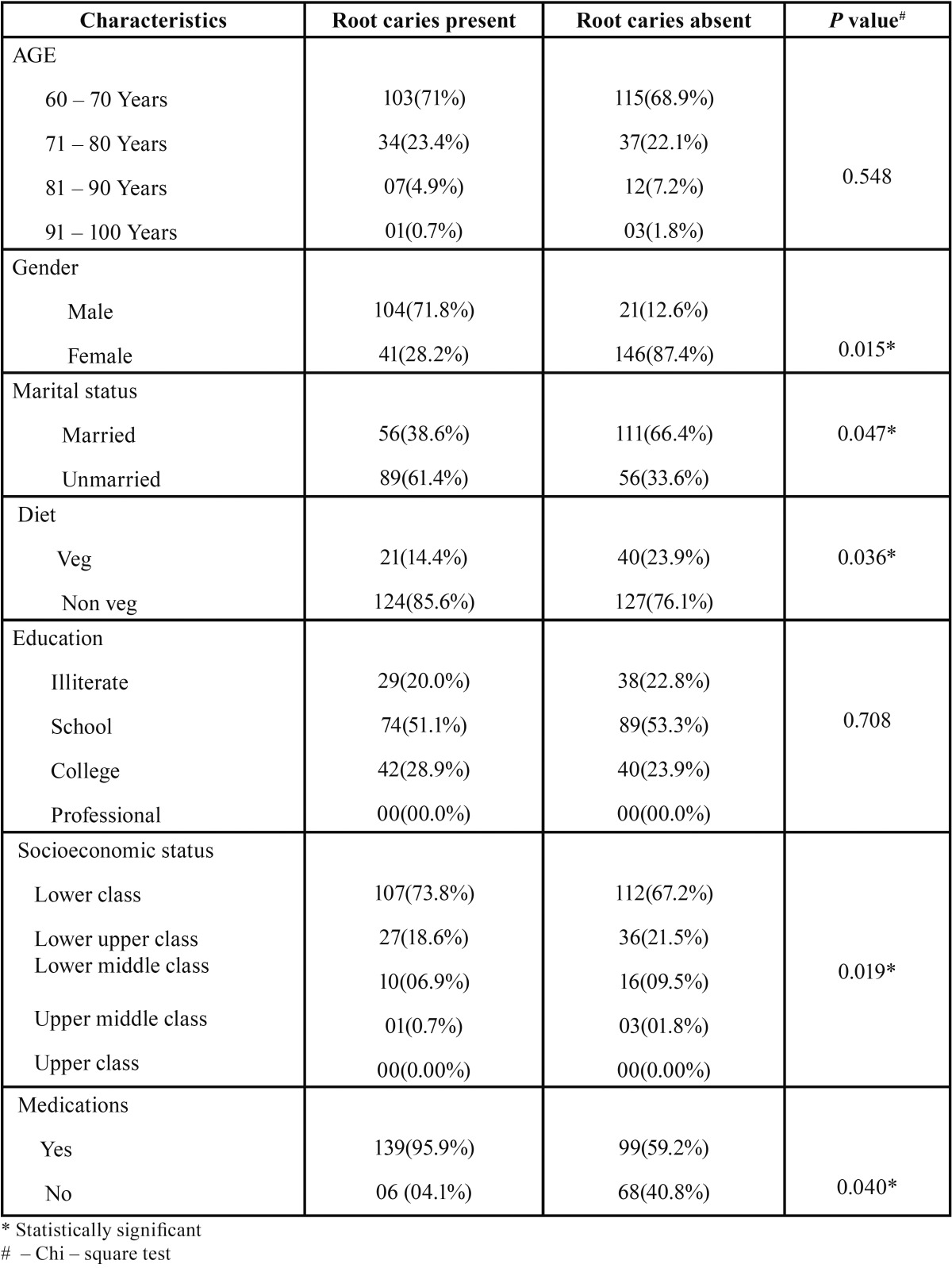
Root caries according to demographic characteristics.

Socioeconomic status (SES) is an economic and sociological combined total measure of a person’s work experience and of an individual’s or family’s economic and social position in relation to others, based on income, education, and occupation. Hence, in the present study Kuppuswamy’s socioeconomic status scale was used in which 219(70.2%) belonged to lower class, 26(8.33%) belong lower middle class, 63(20.2%) belonged to lower upper class, 03(0.96%) belonged to middle class and 02(0.64%) belonged to upper class. Medications were used by 238(76.3%) of study participants and 251(80.4%) had a mixed diet.

In the study group, 308(98.7%) brushed with toothbrush and 04(1.3%) brushed with finger, in which 305(97.7%) subjects brushed once daily, 07(2.3%) brushed twice daily. Majority of the subjects 305(97.7%) brushed in horizontal method, and 08(02.6%) subjects had a habit of mouth rinsing after every meal, 149(47.8%) occasionally rinsed their mouth and 155(49.6%) did not had a habit of mouth rinsing ( Table 2).

**Table 2:**
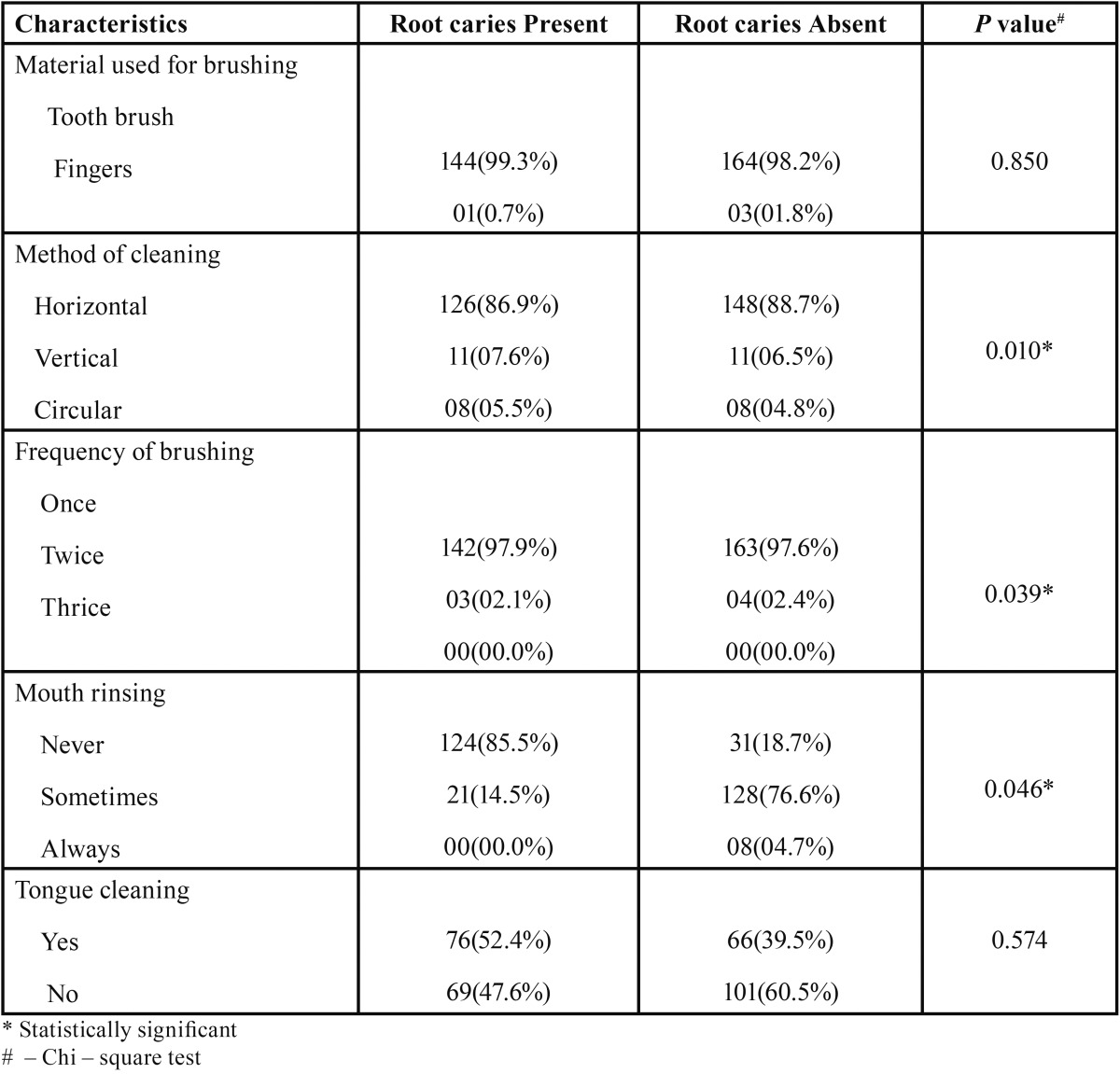
Root caries and oral hygiene habits.

Regarding oral health habits, 62(19.8%), 52(16.6%) and 51(16.3%) had a habit of smoking, pan chewing and alcohol consumption, respectively ( Table 3).

**Table 3:**
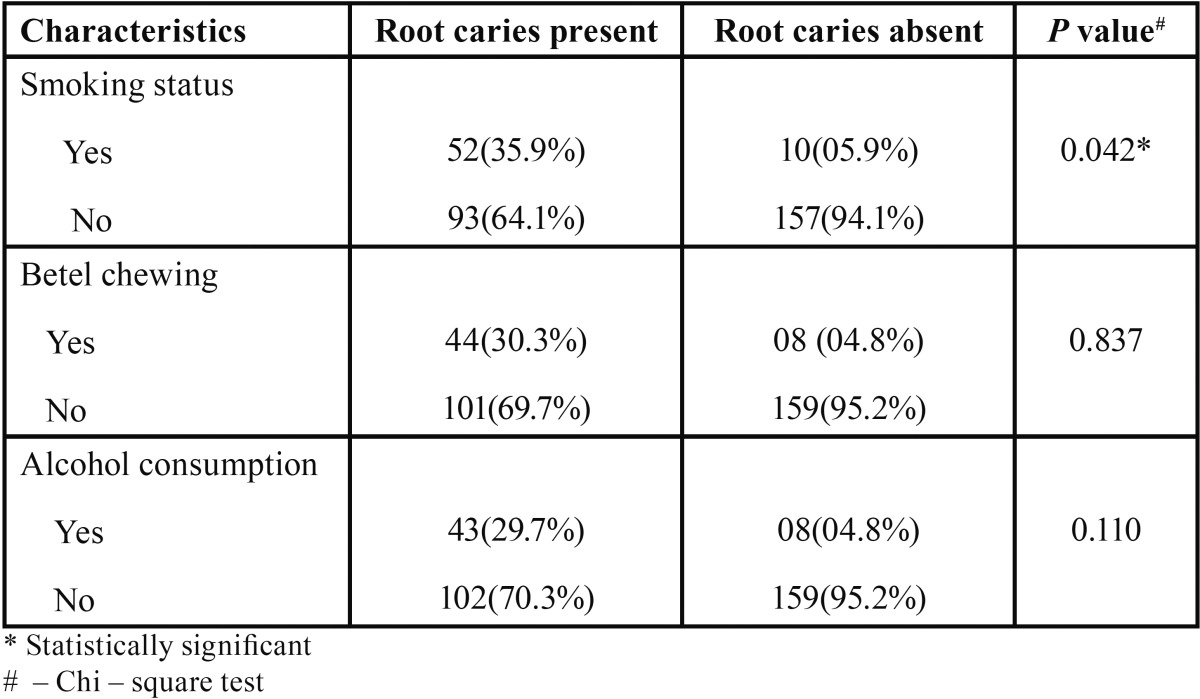
Root caries and oral health behaviours.

Of the several variables that were considered in the bivariate analysis, significant predictors of root surface caries were considered for multiple logistic regression analysis: gender, diet, socio economic status, medications, method of cleaning and frequency of brushing were found to significant predictors of root caries ( Table 4).

**Table 4:**
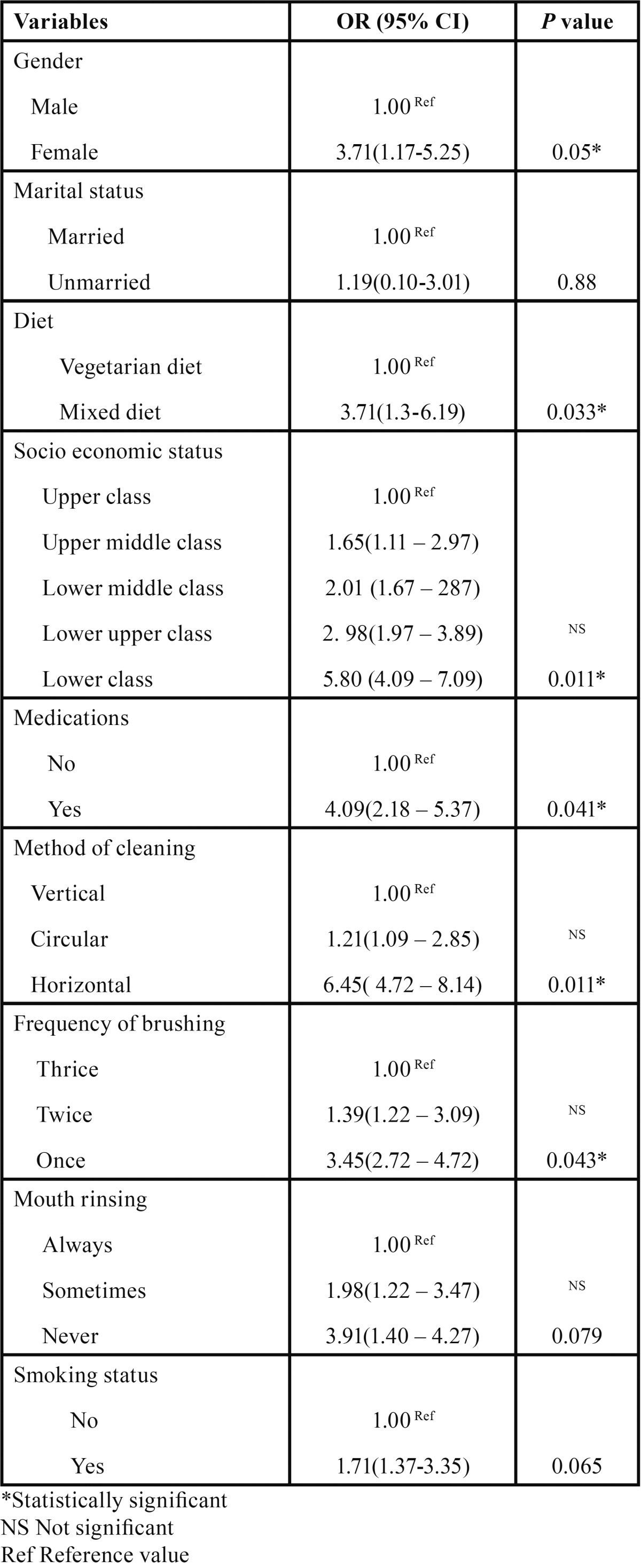
Associations of demographic factors, oral hygiene habits, and oral health behaviour with root caries.

The ratio of the number of teeth with carious lesions on the root and restorations of the root to the number of teeth with exposed root surfaces is known as root caries index (RCI). Overall RCI was higher in relation to maxillary arch than mandibular arch. In both arches, the molar teeth were the most affected by root surface caries. RCI values was more in maxillary left first molar (33.3%) followed by maxillary left second molar (24%) and second left premolar (23%). Lower values of RCI were found in maxillary right third molar (3.20%) followed by lower right third molar (3.69%) and left maxillary lateral incisors (6.97%) ( Table 5).

**Table 5:**
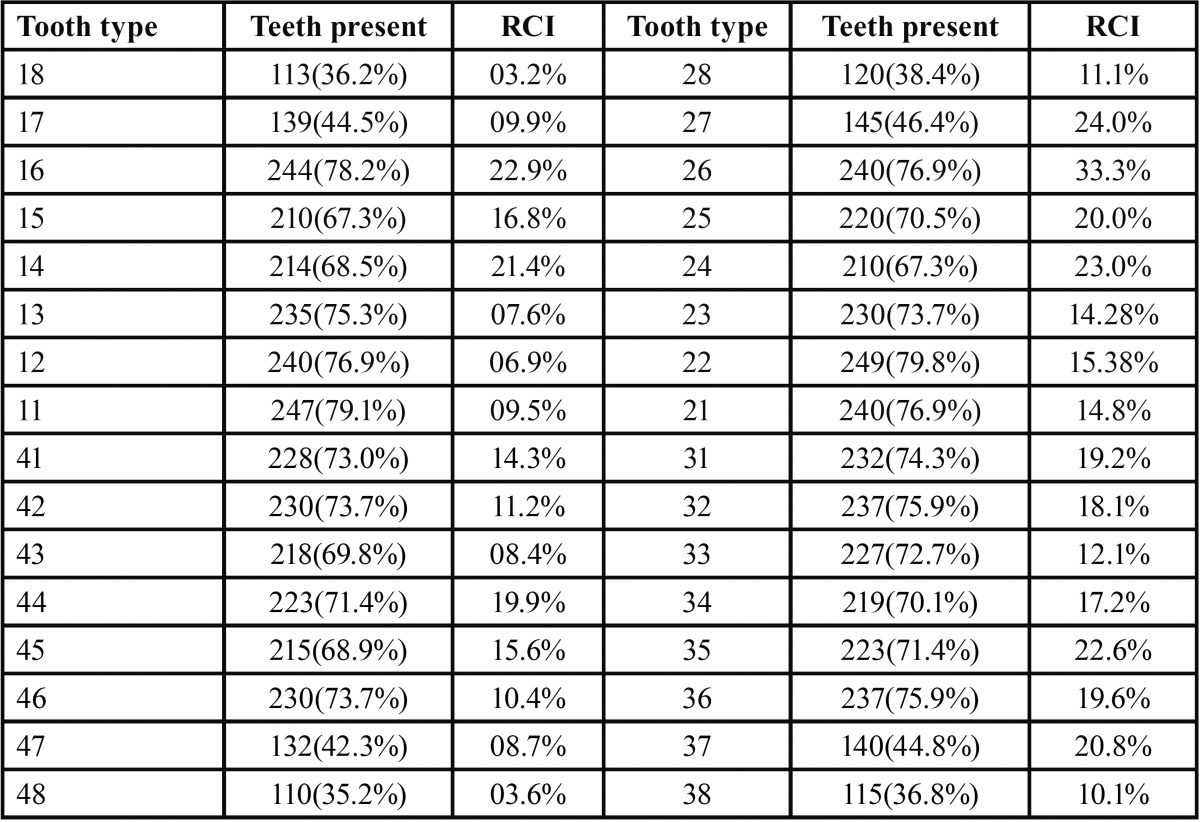
Number of teeth present and RCI for individual teeth in both arches.

## Discussion

The present study provides much-needed data for elderly individuals in India. Root surface decay prevalence and RCI scores were all used to evaluate survey results.

The prevalence of root caries was found to be 46.4% in the present study, which was similar to results of Imazato *et al.* (8) and Bharateesh *et al.* (9) but was in contrast with studies done by Kularatne *et al.* (10) and Watanabe (11). In terms of the Root Caries Index (RCI), which expresses the risk of developing caries due to the root surface being exposed to the buccal environment, the observed value among the study participants was 15.1%, which means that 15% of the root surface are at risk of developing caries per person which was similar to the results given by Kularatne *et al.* (10) and Steele *et al.* (12) and contrast to Splieth *et al.* (13).

When analyzing the RCI according to teeth, the larger scores were observed in the molars and premolars of the maxillary arch and the smaller scores in the mandibular third molar, which was in contrast to findings of Watanabe *et al.* (14) and Fejerskov *et al.* (15).

Many studies in the past literatures had found that gingival recession was a main risk factor for root caries (9); 80.1% of the study participants had gingival recession in one or more teeth, which was similar to our study.

The present study showed that root caries varied by age, and it was more frequent as age advances; however no statistical association was found, which was similar to studies done by Hintao *et al.* (16) and Marlow *et al.* (17). In contrast Sugandhi *et al.* (18) found root caries increased with the increasing age and considered the increasing prevalence of root decay with age might not be due to aging but might be a product of the general deterioration in oral health which often accompanies growing old and also considered physical incapability, psychological impairment and less motivation as a main reasons for root caries development as age advances.

Subjects who were married had a less prevalence of root caries when compared to unmarried subjects, which was found to be statistically significant (*p* = 0.047*). It was considered that living alone seems to have an influence on the disease. It is known that elders who live with a partner have better overall health status than those living alone or with no partner (19).

A statistically significant difference was observed for the presence of root caries among different socioeconomic status (*p* = 0.019*). A wide range of factors have been implicated in caries initiation and progression but these are dominated by the social determinants of health (20) and a low socioeconomic status has been reported to be a risk factor for root caries. Slade *et al.* (21) reported that pre existing socio demographic factors prior to institutionalization were responsible for the higher probability of oral diseases.

Medications that are prescribed to the elderly in fact can cause impaired salivary flow with no change in the immune system (22). Many medications, chemotherapy, radiation treatments, and some diseases can decrease salivary gland function, and therefore make caries and other oral diseases more likely to occur. Some common drugs that may cause dry mouth are high blood pressure drugs, cholesterol lowering drugs, pain medications, muscle relaxants, allergy, and asthma medications (23).

The prevalence of root caries was more among tobacco chewers and smokers, and the results were found to be statistically significant. This finding was in agreement with studies done by Tomar *et al.* (24) and Fure (25). Robertson (26) considered that the use of smokeless tobacco increased the prevalence of gingival recession with associated attachment loss, cervical abrasion and root caries.

The present study also showed presence of root caries among betel chewers which was contrast to study done by Kularatne *et al.* (10) where they found that betel chewing was negatively associated with development of root surface caries. A plausible explanation reported was betel stains on root surfaces act as a chemical or physical barrier against acid attack. Moreover, Reichart *et al.* (27) also reported that *Streptococcus mutans* was not found in betel chewers. This suggests that betel has antibacterial properties that may protect individuals from dental caries which was not evident in the present study.

Hence, prevention of occurrence of root caries may be difficult because root caries often arises in older people who are otherwise also having problems in maintaining good levels of oral hygiene. In addition, older people are frequently taking medication which depresses salivary flow and this xerostomia makes dental caries more likely to occur. The feeling of a dry mouth may be alleviated by sucking sweets or taking frequent drinks, many of which are cariogenic (28). Maintenance or improvement of oral hygiene is the first step towards prevention. Specific measures like the use of powered toothbrushes and chemical plaque control may be advocated. Addition of fluorides to the daily use oral hygiene aids like toothpastes and the use of chlorhexidine gluconate have also shown promising results (29). Finally primordial prevention of root caries lies in the prevention of gingival and periodontal disease (28).

-Limitations

• Despite including a large and representative sample of elderly, this study cannot yield conclusive data on causality because of its cross-sectional design. So, longitudinal studies will be required to further address the link.

• Dental care utilization which was not analysed in this study may pose a major contributing risk factor for root caries and future studies are needed to address this relationship.

-Recommendations 

1. In view of depressingly low levels of oral health in residents, it is important to integrate the preventive measures and appropriate actions can be implemented.

2. Oral health should be incorporated into routine assessment by care staff, and the continuing dental care should be available to the residents.

3. There should be an easy access and timely approach to general and specialist dental services and to oral hygiene equipment appropriate to individual needs of residents.

## Conclusions

In conclusion, results of this oral epidemiological survey showed that the prevalence of root caries among institutionalized older people in Bengaluru city, India was high. Oral health policies and preventive measures are needed focusing on the special needs of this neglected and socioeconomically deprived population to improve their quality of life. Moreover gender, marital status, diet, socio-economic status, medication, method of cleaning and frequency of cleaning were identified as significant predictors of root caries.
